# ﻿Factors related to species richness, endemism, and conservation status of the herpetofauna (Amphibia and Reptilia) of Mexican states

**DOI:** 10.3897/zookeys.1097.80424

**Published:** 2022-04-20

**Authors:** Geoffrey R. Smith, Julio A. Lemos-Espinal

**Affiliations:** 1 Department of Biology, Denison University, Granville, Ohio 43023, USA Denison University Granville United States of America; 2 Laboratorio de Ecología-UBIPRO, FES Iztacala UNAM, Avenida los Barrios 1, Los Reyes Iztacala, Tlalnepantla, edo. de México, 54090, Mexico FES Iztacala UNAM Tlalnepantla Mexico

**Keywords:** amphibians, environmental factors, human demographic factors, IUCN status, reptiles, SEMARNAT listing, socioeconomic factors

## Abstract

Mexico is a megadiverse country with high endemicity in its herpetofauna. We examine how species richness, proportion of state and country endemic species, and proportion of species in a category of conservation concern using listings in the International Union for Conservation of Nature (IUCN) Red List and the Secretaría del Medio Ambiente y Recursos Naturales (SEMARNAT) in 27 of 32 Mexican states are related to environmental and human demographic and socioeconomic variables. Amphibian and reptile species richness were positively related to latitude range and number of physiographic regions and negatively related to latitude. The proportion of state endemic amphibian species in a state was negatively related to latitude whereas no variables influenced the proportion in reptiles. The proportion of country endemics in a state was positively related to human population density and the number of physiographic regions and negatively related to per capita gross domestic product (GDP) and latitude range for amphibians; it was positively related to human population density and elevation range and negatively related to latitude range for reptiles. The proportion of amphibian species in an IUCN category of concern in a state was positively related to human population density and negatively related to latitude; for reptiles, it was negatively related to human population density. The proportion of SEMARNAT-listed species in a state was positively related to human population density for both amphibians and reptiles and negatively related to latitude range for amphibians. Our analyses found that larger macroecological patterns (e.g., latitudinal species gradient, heterogeneity-richness relationships) and human population density play important roles in determining the richness and conservation status of Mexican amphibians and reptiles.

## ﻿Introduction

Much of Mexico consists of a transition zone (i.e., the Mexican Transition Zone) between the Nearctic and Neotropical zones (Villaseñor et al. 2020). As a consequence, Mexico has one of the highest levels of biodiversity of any country in the world (Morrone 2019), including amphibian and reptile richness (Wilson and Johnson 2010; Chen and Peng 2017). Mexico also has a high level of endemicity in its herpetofauna (Wilson and Johnson 2010; see also Murali et al. 2021).

Unfortunately, Mexico is not immune to global environmental decline. Indeed, Mexico is an area with high extinction debt and risk for amphibians and reptiles (Chen and Peng 2017). In addition, Mexico is one of the countries where the decline in the conservation status of amphibians is greatest (Rodrigues et al. 2014) and has a high proportion (ca 80%) of species of amphibians showing population declines (Becker and Loyola 2008). The biodiversity of Mexico is subject to a variety of pressures, such as climate change, land use change (including agriculture and livestock, mining, deforestation, and urbanization), invasive species, disease, exploitation, and pollution (Rodrigues et al. 2014; Lazcano et al. 2019; Ramírez-Bautista et al. 2020; González-Sánchez et al. 2021; Masés-García et al. 2021), which are the consequence of human demographic changes (e.g., population growth), socioeconomics, and governmental actions (Challenger et al. 2009; Sarukhán et al. 2015).

Human pressure on biodiversity has increased in the Neotropics (Geldmann et al. 2014). More specifically, mean annual temperatures in Mexico have increased around 0.2 °C from 1970–2000, with greater increases in northern Mexico than in southern Mexico (see also Pavia et al. 2009; Cuervo-Robayo et al. 2020). This climate change has driven changes in the vegetation and distribution of habitats, especially in the mountains of Mexico (Téllez-Valdés et al. 2006; Gómez-Mendoza and Arriaga 2007; Jiménez-García et al. 2021), and continued climate change is likely to result in the loss of suitable habitat in the future (Chacón-Prieto et al. 2021). Parts of Mexico are also undergoing rapid land use change through burning, human settlement, and conversion to agriculture, with extensive loss of forest, including in protected areas (Lorenzo et al. 2017; Hu et al. 2021). Habitat loss has impacted several species of terrestrial vertebrates in Mexico, especially endemic species in the Transvolcanic Mexican Belt, Mexican High Plateau, and the Humid Coastal Plains and Hills of the Gulf of Mexico, and the effects appear to be cumulative (i.e., not just recent habitat loss) (Mayani-Parás et al. 2021). The loss of habitat, and in particular forest habitats, has negatively affected the amphibians and reptiles of Mexico (Lara-Tufiño et al. 2019; Mayani-Parás et al. 2019), and a high proportion of endangered amphibians in Mexico are found in areas that have experienced transformation to agriculture or urbanization (Londoño-Murcia and Sánchez-Cordero 2011) and these trends are likely to continue or increase in the future (Mendoza-Ponce et al. 2020).

Here we examine the distribution of amphibian and reptile species richness among 27 of 32 Mexican states. In particular, we examine relationships between species richness, proportion of state and country endemic species (i.e., the proportion of the species in a state that are state or country endemics), and proportion of a state’s amphibian or reptile species in a category of conservation concern using the Interational Union for Conservation of Nature (IUCN) Red List (i.e., vulnerable, threatened, endangered, critically endangered, near extinction), and the proportion of a state’s amphibian or reptile species listed in Secretaría del Medio Ambiente y Recursos Naturales (SEMARNAT) (2019) with environmental variables (state area, proportion of land protected, latitude, latitude range, elevation range, and number of physiographic regions) and human demographic and socioeconomic variables [human population, human population density, and per capita gross domestic product (GDP)].

## ﻿Methods

We collected species lists for amphibians and reptiles of Mexican states from the available literature and updated these species lists using additional literature through November 2020 (see Suppl. material 1: Table S1 for sources used for base species lists and updates). We generally followed Frost (2020) and AmphibiaWeb (2020) for amphibian taxonomy and Uetz and Hošek (2019) for reptile species. We were able to compile updated species lists for 27 of the 32 Mexican federal entities (i.e., states), with the five remaining states lacking published updated species checklists (Suppl. material 2: Table S2). We include Mexico City (formerly known as Mexico, Distrito Federal) that comprises the urban area of Mexico City proper to the south and mountains and valleys with fragmented forests and grasslands to the north. For each species in our list, we obtained their global conservation status from the IUCN Red List version 2021-3 (https//:www.iucnredlist.org) and their Mexican conservation status from SEMARNAT (2019) (Suppl. material 2: Table S2). From these lists we gathered the following information for each state: species richness, proportion of state and country endemics, proportion of species in an IUCN category of concern (critically endangered, endangered, threatened, near threatened, and vulnerable), and the proportion of SEMARNAT-listed species under the categories of threatened and endangered for amphibians and reptiles separately (Table 1). For each state we collected data on human demographic and socioeconomic variables (human population, human population density, and per capita GDP) and geographic and climatic variables [state area, proportion of land protected, latitude (midpoint of state), latitude range (difference between minimum and maximum latitude), elevation range (difference between minimum and maximum elevations), and number of physiographic regions] (Table 2).

**Table 1.** Amphibians and reptile species richness, proportion of species in a state that are state and country endemics, proportion of species that are in an IUCN category of concern, and the proportion of species that are SEMARNAT listed for Mexican states.

State	Amphibians					Reptiles				
	Species Richness	Prop. State Endemic	Prop. Country Endemic	Prop. IUCN	Prop. SEMARNAT	Species Richness	Prop. State Endemic	Prop. Country Endemic	Prop. IUCN	Prop. SEMARNAT
Aguascalientes	19	0	0.68	0.16	0.16	65	0	0.54	0.05	0.15
Baja California	17	0	0	0.29	0.06	103	0.23	0.32	0.21	0.23
Baja California Sur	3	0	0	0	0	84	0.44	0.57	0.13	0.32
Campeche	24	0	0.04	0	0	103	0	0.12	0.13	0.16
Chiapas	108	0.10	0.21	0.53	0.04	219	0.06	0.18	0.11	0.16
Chihuahua	37	0.03	0.35	0.11	0.03	140	0.01	0.33	0.06	0.15
Coahuila	24	0	0.25	0.17	0.04	117	0.06	0.30	0.15	0.22
Colima	39	0.03	0.68	0.13	0.08	117	0.04	0.66	0.11	0.18
Durango	36	0	0.6	0.11	0.03	119	0.02	0.45	0.05	0.18
Guerrero	78	0.32	0.76	0.45	0.11	181	0.15	0.68	0.08	0.16
Hidalgo	52	0.08	0.65	0.46	0.15	126	0.01	0.49	0.10	0.18
Jalisco	55	0.06	0.72	0.22	0.07	173	0.01	0.66	0.10	0.17
Mexico	49	0.08	0.77	0.42	0.21	99	0.01	0.71	0.08	0.17
Mexico City	18	0.11	0.83	0.5	0.39	45	0	0.76	0.04	0.22
Michoacán	58	0.09	0.74	0.26	0.09	161	0	0.7	0.09	0.18
Morelos	38	0	0.70	0.27	0.14	97	0	0.68	0.04	0.17
Nayarit	37	0	0.6	0.08	0.03	117	0.01	0.60	0.07	0.14
Nuevo León	25	0	0.32	0.2	0.04	119	0.02	0.34	0.09	0.20
Oaxaca	152	0.39	0.69	0.60	0.10	299	0.11	0.55	0.11	0.18
Puebla	92	0.03	0.7	0.49	0.16	176	0.02	0.61	0.07	0.14
Querétaro	34	0.60	0.56	0.32	0.12	104	0.02	0.49	0.07	0.18
Quintano Roo	23	0	0.09	0.04	0	108	0.01	0.12	0.12	0.16
San Luis Potosí	42	0	0.41	0.34	0.12	138	0	0.41	0.09	0.17
Sinaloa	39	0	0.55	0.13	0	119	0.01	0.51	0.10	0.14
Sonora	36	0	0.39	0.11	0.03	159	0.10	0.37	0.13	0.18
Tamaulipas	44	0.11	0.41	0.27	0.14	137	0.04	0.34	0.11	0.21
Yucatán	17	0	0.12	0.06	0	85	0	0.12	0.12	0.18

**Table 2.** Human demographic and socioeconomic variables and environmental variables for Mexican states.

State	State Area (km^2^)^1^	Human Population (2018)^1^	Human Population Density (N/km^2^)^1^	Per capita GDP (US$)^2^	Proportion Protected Territory^3^	GPS Coordinates^1^ (°)	Elevation Range (m)^1^	Number of Physiographic Regions^4^
Aguascalientes	5618	1.337,792	238.1	9975	26.0	22.1243, 1.0042	1666	3
Baja California	71.450	3.633,772	50.9	9449	19.0	30.3593, 4.7186	3100	2
Baja California Sur	73.909	832.827	11.3	11.060	42.0	25.4360, 5.1280	2080	1
Campeche	57.507	948.459	16.5	51.460	39.6	19.6167, 0.7667	390	2
Chiapas	73.311	5.445,233	74.3	3592	18.0	17.2588, 5.4530	4080	3
Chihuahua	247.460	3.816,865	15.4	8833	8.1	28.6843, 6.4175	3050	2
Coahuila	151.595	3.063,662	20.2	12.838	19.0	27.2114, 5.3372	3380	3
Colima	5627	759.686	135	9177	6.6	19.0983, 0.8283	3820	2
Durango	123.317	1.815,966	14.5	7888	22.1	24.5950, 4.5000	3240	4
Guerrero	63.596	3.625,040	5.7	4586	0.15	17.6018, 2.5719	3550	2
Hidalgo	20.813	2.980,532	143.2	6508	6.9	20.4982, 1.8008	3251	3
Jalisco	78.588	8.197,483	104.3	9239	11.2	20.8380, 3.8244	4339	4
Mexico	22.500	17.604,619	782.4	6199	43.8	19.3264, 1.9189	5268	2
Mexico City	1495	8.788,141	5878.4	21.079	14.1	19.3206, 0.5444	1702	1
Michoacán	58.599	4.687,211	80	5522	5.9	19.1547, 2.4794	4100	2
Morelos	4879	1.987,596	407.4	6961	26.8	18.7319, 0.7994	4580	2
Nayarit	27.857	1.290,519	46.3	6220	30.8	21.8439, 2.4811	2760	4
Nuevo León	64.156	5.300,619	82.6	16.228	8.9	25.4810, 4.6364	3660	3
Oaxaca	93.757	4.084,674	43.6	4446	7.1	17.1635, 3.0125	3720	5
Puebla	34.306	6.371,381	185.7	5890	19.5	19.3500, 2.9667	5530	4
Querétaro	11.699	2.091,823	178.8	12.502	33.6	20.8425, 1.655	2600	3
Quintano Roo	50.212	1.709,479	34	11.381	32.6	19.7000, 3.7667	230	1
San Luis Potosí	61.137	2.824,976	46.2	8118	6.6	22.8258, 3.3311	3160	3
Sinaloa	58.328	3.059,322	52.5	8108	7.6	24.7547, 4.5750	2520	2
Sonora	179.355	3.050,473	17	11.543	10.3	29.3954, 6.1969	2620	4
Tamaulipas	80.249	3.661,162	45.5	9347	13.7	24.9430, 5.4722	3280	3
Yucatán	39.524	2.199,618	55.7	8.011	25.6	20.5667, 2.0667	210	1

^1^INEGI (2018) ^2^https://es.wikipedia.org/wiki/Anexo:Estadios_de_M%C3%A9xico_por_PIB_per_c%C3%A1pita^3^http://sig.conanp.gob.mx/website/pagsig/listanp/^4^https://www.monografias.com/trabajos100/regions-fisiograficas-mexico/regions-fisiograficas-mexico.shtml#llanurasoa

We used generalized linear models (Normal distribution, Identity link) for amphibians and reptiles separately to examine the relationships of the human demographic and socioeconomic variables and the geographic and climatic variables and species richness, proportion of country endemics, proportion of state endemics, proportion of species in an IUCN category of concern, and proportion SEMARNAT-listed species. We used JMP Pro 15.1 (SAS Institute, Cary, NC). for statistical analyses.

## ﻿Results

**Figure 1.**
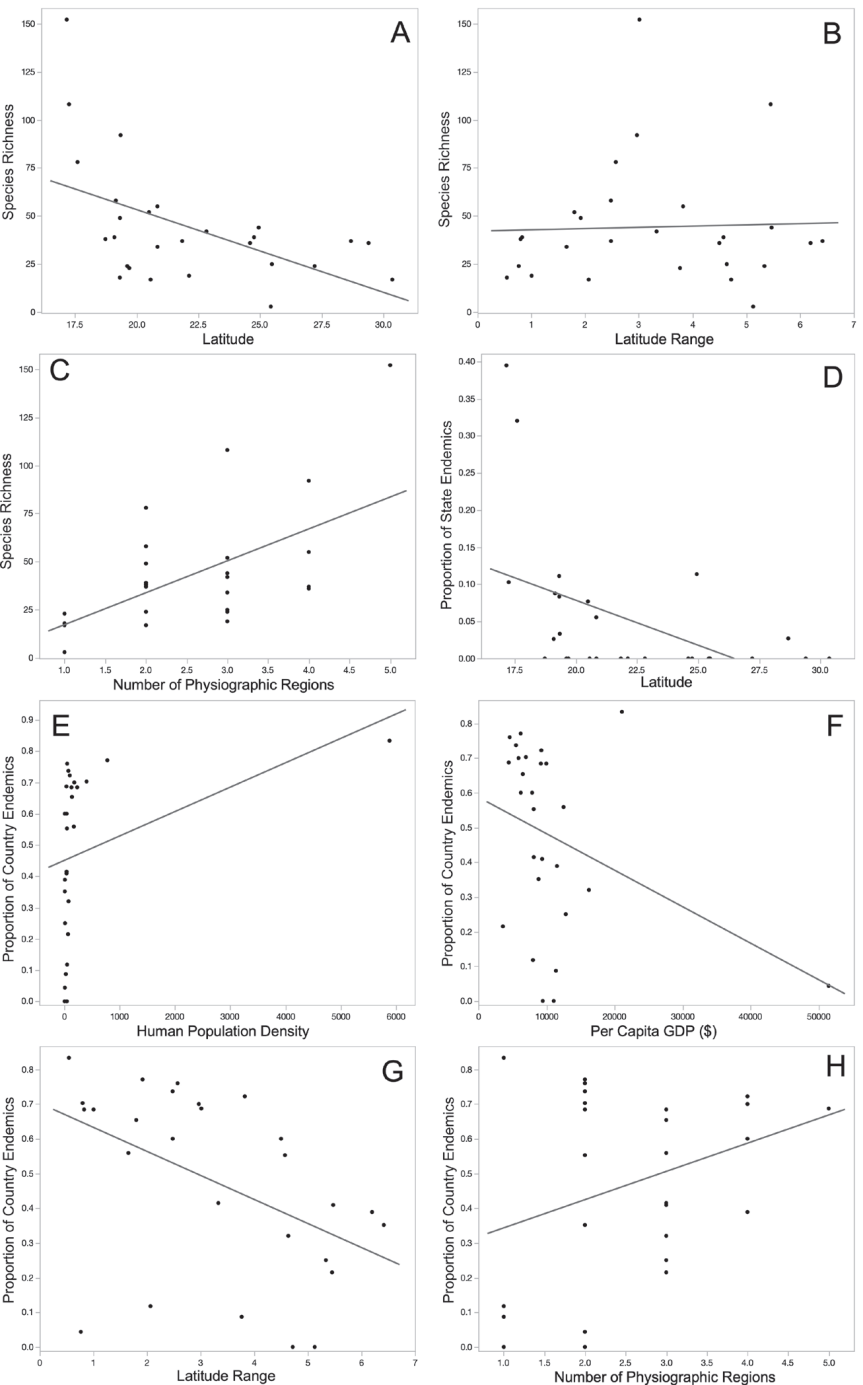
The relationships between amphibian species richness and a state’s latitude **A** latitude range **B** and number of physiographic regions **C** between the proportion of a state’s amphibian species that are state endemics and a state’s latitude **D** and between the proportion of a state’s amphibian species that are country endemics and the state’s human population density **E** per capita GDP**F** latitude range **G** and the number of physiographic regions for Mexico **H**.

**Figure 2.**
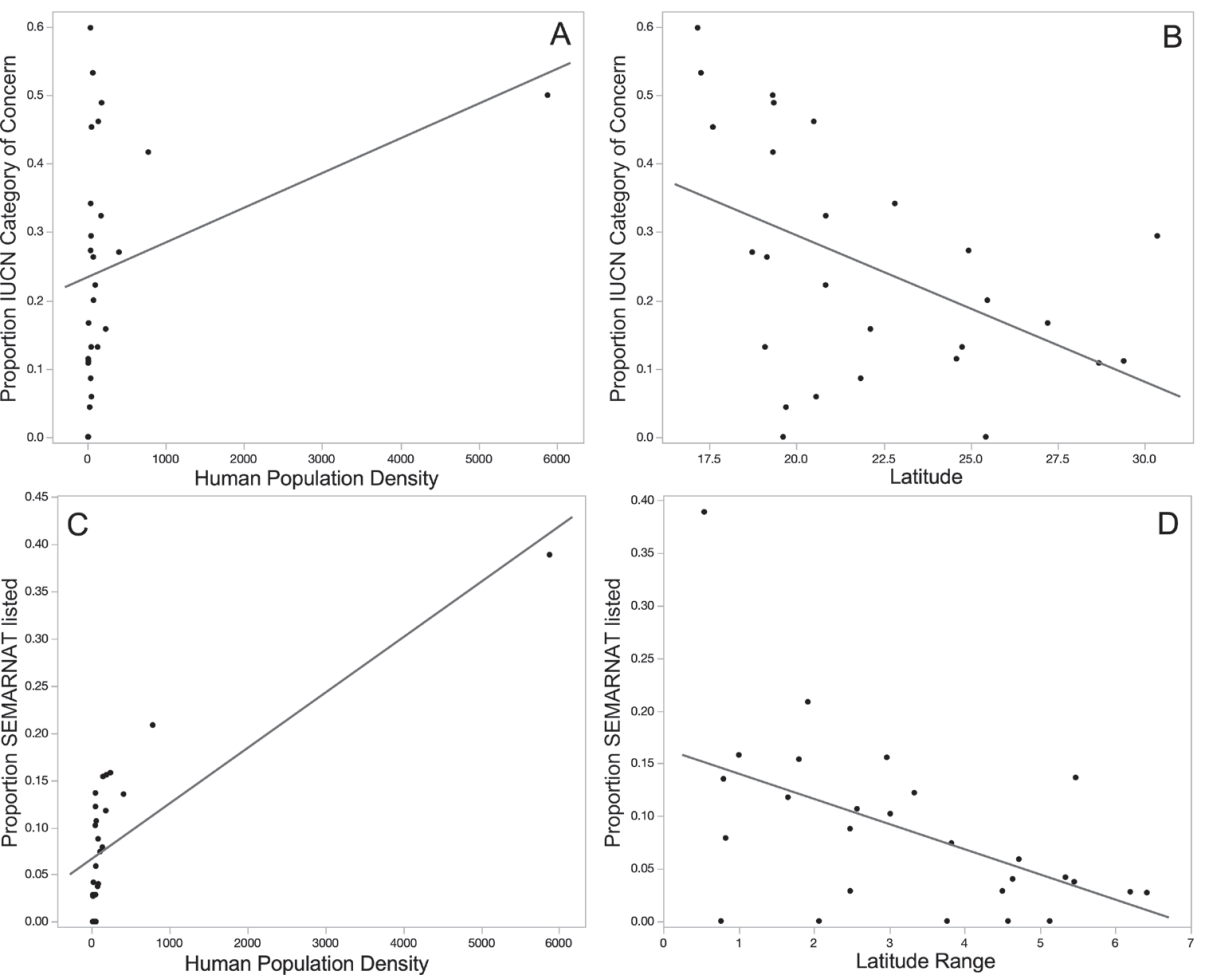
The relationships between the proportion of a state’s amphibian species that are in an IUCN category of concern and a state’s human population density **A** and latitude **B** and between the proportion of a state’s amphibian species that are listed in SEMARNAT (2019) and a state’s human population density **C** and latitude range for Mexico **D**.

Amphibian species richness was positively related to latitude range and number of physiographic regions and negatively related to latitude (Table 3; Fig. 1A-C). The proportion of state endemics was negatively related to latitude (Table 3; Fig. 1D). The proportion of country endemics was positively related to human population density and the number of physiographic region and negatively related to per capita GDP and latitude range (Table 3; Fig. 1E-H). The proportion of amphibian species in an IUCN category of concern was positively related to human population density and negatively related to latitude (Table 3; Fig. 2A, B). The proportion of SEMARNAT-listed amphibian species was positively related to human population density and negatively related to latitude range (Table 3; Fig. 2C, D).

**Table 3.** Results of generalized linear models examining the relationship between human demographic and socioeconomic variables and environmental variables and species richness, proportion of species in a state that are state and country endemics, proportion of species that are in an IUCN category of concern, and the proportion of species that are SEMARNAT listed for amphibians in 27 Mexican states. Values on first line are coefficients, values on second line are *P*-values. Bolded entries are significant at α = 0.05.

Variable	Intercept	State area	Human population	Human population density	per capita GDP	Proportion Protected	Latitude	Latitude Range	Elevation range	Physiographic regions	Overall P
Species Richness	**153.03 < 0.0001**	0.00011 0.16	< 0.00001 0.63	-0.0010 0.725	-0.0002 0.18	-44.61 0.074	-**7.37 < 0.0001**	**5.91 0.043**	0.0025 0.39	**10.62 0.0006**	**< 0.0001**
Prop State endemic	**0.447 0.0003**	< 0.000001 0.17	<0.0000001 0.39	<0.000001 0.65	-<0.000001 0.49	-0.247 0.057	-**0.018 0.0009**	0.004 0.81	-<0.00001 0.52	0.013 0.36	**0.0108**
Prop Country endemic	**0.568 0.0046**	0.000001 0.10	<<0.000001 0.36	**0.000062 0.024**	-**0.0000010 0.004**	-0.41 0.066	0.00055 0.95	-**0.116 < 0.0001**	0.000047 0.078	**0.069 0.008**	**<0.0001**
Prop IUCN	**0.469 0.0065**	-<0.000001 0.35	<<0.000001 0.26	**0.000050 0.0336**	-0.0000016 0.55	-0.33 0.086	-**0.018 0.017**	0.022 0.30	0.000029 0.20	0.035 0.10	**0.0002**
Prop SEMARNAT	0.0045 0.93	-0.0000001 0.56	<<0.0000001 0.14	**0.000050 < 0.0001**	-0.0000012 0.19	-0.042 0.19	0.0034 0.18	-**0.018 0.016**	0.000012 0.12	0.0096 0.19	**<0.0001**

**Figure 3.**
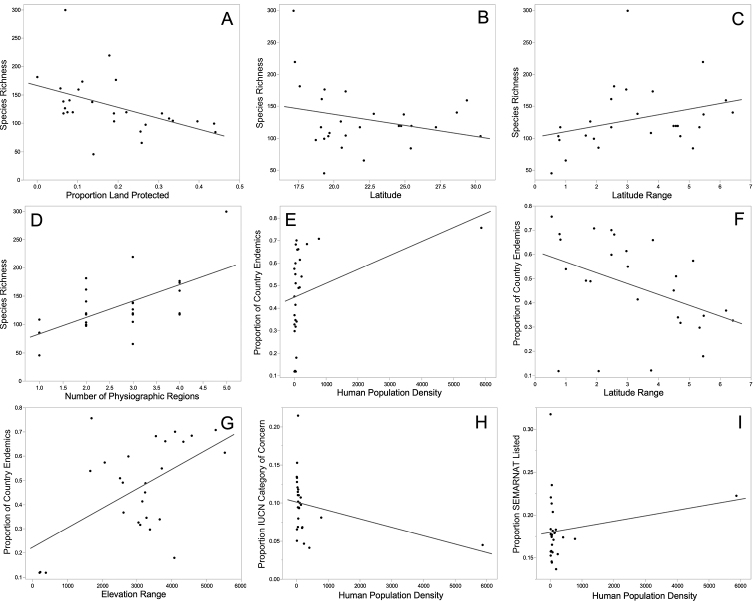
The relationships between reptile species richness and a state’s proportion of protected land **A** latitude **B** latitude range **C** and number of physiographic regions **D** between the proportion of a state’s reptile species that are country endemics and the state’s human population density **E** latitude range **F** and elevation range **G** between the proportion of a state’s reptile species that are in an IUCN category of concern and a state’s human population density **H** and between the proportion of a state’s reptile species that are listed in SEMARNAT (2019) and a state’s human population density for Mexico **I**.

Reptile species richness was positively related to latitude range and the number of physiographic regions and negatively related to latitude and the proportion of land protected (Table 4; Fig. 3A-D). The proportion of state endemics of reptiles was not related to any variables (Table 4). The proportion of country endemic reptile species was positively related to human population density and elevation range and negatively related to latitude range (Table 4; Fig. 3E-G). The proportion of a state’s reptile species in an IUCN category of concern was negatively related to human population density (Table 4; Fig. 3H). The proportion of a state’s reptile species that are SEMARNAT listed was positively related to human population density (Table 4; Fig. 3I).

**Table 4.** Results of generalized linear models examining the relationship between human demographic and socioeconomic variables and environmental variables and species richness, proportion of species in a state that are state and country endemics, proportion of species that are in an IUCN category of concern, and the proportion of species that are SEMARNAT listed for reptiles in 27 Mexican states. Values on first line are coefficients, values on second line are *P*-values. Bolded entries are significant at α = 0.05.

Variable	Intercept	State area	Human population	Human population density	per capita GDP	Proportion protected	Latitude	Latitude Range	Elevation range	Physiographic regions	Overall P
Species Richness	**279.65 < 0.0001**	0.00023 0.06	0.0000005 0.73	-0.0084 0.061	0.00026 0.62	-**109.7 0.0046**	-**10.74 < 0.0001**	**13.35 0.0031**	0.0013 0.76	**16.57 0.0003**	**<0.0001**
Prop State endemic	-0.070 0.58	-0.0000002 0.61	<0.0000001 0.074	0.000015 0.40	-<0.00001 0.79	0.272 0.072	0.0009 0.87	0.031 0.08	0.000025 0.17	-0.024 0.15	0.22
Prop Country endemic	0.245 0.18	0.00000035 0.61	<0.0000001 0.39	**0.000068 0.011**	-0.0000065 0.039	0.082 0.69	0.0094 0.26	-**0.067 0.009**	**0.000091 0.0011**	-0.0016 0.95	**0.0002**
Prop IUCN	0.051 0.28	-0.0000003 0.095	<0.0000001 0.34	-**0.000015 0.035**	0.0000014 0.10	-0.064 0.24	0.0024 0.28	0.0122 0.063	-0.000005 0.42	-0.0060 0.34	0.076
Prop SEMARNAT	0.079 0.087	-0.0000002 0.23	-<<0.00001 0.084	**0.000015 0.028**	0.00000025 0.74	0.088 0.10	0.0031 0.14	0.010 0.09	0.000011 0.10	-0.010 0.083	0.054

## ﻿Discussion

Our analyses found that species richness, endemism, and conservation status of amphibians and reptiles in Mexican states are related to both human demographic and socioeconomic variables and environmental variables. Below we discuss our observations on the factors related to species richness, endemism, and conservation status of amphibians and reptiles in Mexico.

### ﻿Species richness and endemicity

For amphibians, species richness was positively related to latitude range and the number of physiographic regions in a state and negatively related to latitude (i.e., species richness decreased with latitude), whereas reptile species richness was positively related to latitudinal range and the number of physiographic regions and negatively related to latitude and the proportion of land protected. The proportion of a state’s amphibian species that are state endemics was negatively related to latitude whereas none of the state variables we examined were related to the proportion of a state’s reptile species that are state endemics. For the proportion of a state’s species of amphibians that are country endemics there was a positive relationship with human population density and the number of physiographic regions and a negative relationship with per capita GDP and latitude range. For reptiles, this proportion was positively related to human population density and elevation range and negatively related to latitude range.

The positive relationships between species richness and latitude range and the number of physiographic regions for both amphibians and reptiles likely result from the increased variety of habitats and climates in a state leading to a greater number of niches, which can result in increased number of species occurring in a state. Our results for the herpetofauna of Mexico are similar to studies on *Sceloporus* lizards (Rivera et al. 2021) and on mammals finding that species richness at a variety of geographic scales increases with habitat or environmental heterogeneity (Amori et al. 2013, 2019; Udy et al. 2021).

The decrease in species richness of amphibians and reptiles with increasing latitude is consistent with the latitudinal species gradient (Pianka 1966; Willig et al. 2003; Hillebrand 2004; Pontarp et al. 2019). Species richness of amphibians in Mesoamerica is highest to the south (Wilson and Johnson 2010). Similarly, mammalian species richness in Mexico decreased with increasing latitude (Ceballos et al. 1998). These results are also similar to those found for amphibians and reptiles in a variety of regions and countries, such as Europe (Assunção-Albuquerque et al. 2012), United States (Schall and Pianka 1978), Australia (Schall and Pianka 1978), and North America (Rivera et al. 2021; Whiting and Fox 2021). The latitudinal gradient of species richness in both amphibians and reptiles in Mexico might be related to latitudinal gradients in climate related factors. For example, the species richness of reptiles and amphibians is often related to solar radiation, temperature, precipitation, annual potential and actual evapotranspiration (e.g., Schall and Pianka 1978; Rodríguez et al. 2005; Powney et al. 2010; Kafesh et al. 2020), all of which vary with latitude.

In addition, for reptiles, species richness decreased with increasing human population density and the proportion of the state’s area protected. This relationship suggests there might be a negative impact of human population on species richness, perhaps due to the impact on amphibian and reptile populations. For example, in Europe, climate and human related factors explained 41–42% of variation in species richness of amphibians and reptiles (Assunção-Albuquerque et al. 2012). The species richness of reptiles in Mediterranean France is correlated with climate, elevation, and land use (Barnagaud et al. 2021). Reptile species richness is negatively affected by livestock production and urbanization (Cordier et al. 2021). Thus, the species richness of reptiles appears to be particularly susceptible to human pressures.

The patterns of state endemism that we observed show limited effects of the state variables we examined, with only a negative relationship between latitude and the proportion of a state’s amphibian species being state endemics. In part this general lack of relationships may reflect the artificial nature of state boundaries (see Caveats below) such that states often share such physiographic regions or habitats and so likely share species, even over a small area along borders. For country-level endemism, we found positive relationships with human population density and measures related to habitat heterogeneity (e.g., number of physiographic regions or elevation range) for both amphibians and reptiles. Such relationships likely reflect the high level of endemism found in the Transvolcanic Mexican Belt (Flores-Villela et al. 2010) which is found in an area of high human population density (e.g., Mexico City and its environs). The negative relationship between country-wide endemism and latitude range likely also reflects the smaller states and federal entities found in central and southern Mexico where high levels of endemism are found.

### ﻿Conservation status

Both human demographic and environmental variables affected the proportion of both amphibians and reptiles of conservation concern in a state. The proportion of amphibian species in an IUCN category of concern decreased with latitude and increased with human population density. For SEMARNAT, the proportion of listed species increased with human population density and decreased with latitudinal range. For reptiles, the proportion of a state’s species in an IUCN category of concern decreased with human population density, but the proportion that is SEMARNAT listed increased with human population density. The importance of human population density in determining conservation status is consistent with the impacts that anthropogenic effects on the environment have on amphibians and reptiles. For example, a high proportion of endangered amphibians in Mexico being found in areas that have experienced transformation to agriculture or urbanization (Londoño-Murcia and Sánchez-Cordero 2011). In addition, amphibian species diversity in central Mexico is reduced with the loss of canopy (Lara-Tufiño et al. 2019). Mayani-Parás et al. (2019) demonstrated that many species of amphibians and reptiles in Mexico have suffered major reductions in their distributions, and that this is particularly the result of the combined effects of mining and habitat loss. In addition, reptiles, and, to a lesser extent, amphibians, are subject to illegal trade and collection in Mexico (Masés-García et al. 2021). These patterns in Mexico are also consistent with patterns of conservation status and human pressures at the global and more regional scales for amphibians and reptiles. Globally, amphibian species richness is susceptible to deforestation, timber harvesting and production, and urbanization (Cordier et al. 2021). Reptile species richness is negatively affected by livestock production and urbanization (Cordier et al. 2021). In addition, reptile abundance globally is negatively affected by anthropogenic habitat changes (Doherty et al. 2020). Declines in European amphibians and reptiles were associated with the number of alien species and loss of habitat due to climate change (Falaschi et al. 2019). In China, the distribution of biodiversity loss is driven primarily by climate and anthropogenic sources (Lu et al. 2020). Similarly, in Australia extinction risk in reptiles is related to anthropogenic pressures and proximity to human populations as well as climatic variables (Senior et al. 2021).

### ﻿Caveats

We recognize that our analysis is a snapshot in time of both taxonomic knowledge and conservation status. Species lists are dynamic and changing as new species are described, new localities are found, and populations are extirpated. In addition, conservation status for species, whether at the global (i.e., IUCN) or national (i.e., SEMARNAT) scale, are frequently reassessed. Thus, we realize that our analysis represents our knowledge at the time we generated our species lists and conducted our analyses. However, obtaining a snapshot now will provide a baseline that can be monitored and evaluated as our understanding of taxonomy, species distributions, and conservation status change over time.

We also recognize that by using global and national conservation status we are not taking into account variation in conservation status of species populations in each state such that species may be doing well in some states but poorly in other states. This is a challenge, but unfortunately conservation status at the state level is known for even fewer species than for the national and global measures. In addition, many regulations are focused on, or use, national or global level assessments rather than state level assessments.

Finally, we further recognize that the use of states in our analyses does not necessarily reflect ecological or biogeographical reality (i.e., they are not natural regions). However, given the nature of governmental processes, conservation efforts are usually a function of state or political boundaries and thus we argue that understanding patterns at the state level is pragmatic.

## Supplementary materials

Supplementary material 1 Table S1 Data type Summary of sources for data used. Explanation note This file contains a list of the checklists from which we obtained our species lists for each Mecixan state. It also provides information on sources used to update those checklists. https://binary.pensoft.net/file/675099This dataset is made available under the Open Database License (http://opendatacommons.org/licenses/odbl/1.0/). The Open Database License (ODbL) is a license agreement intended to allow users to freely share, modify, and use this Dataset while maintaining this same freedom for others, provided that the original source and author(s) are credited.Geoffrey R. Smith, Julio A. Lemos-Espinal

Supplementary material 2 Table S2 Data type Occurrences. Explanation note This file contains the data matrix used to generate the data used in our analyses. Basically it is a matrix of presence and absence of herpetofaunal species in each of the considered Mexican states. This matrix was created using the sources provided in Table S1. https://binary.pensoft.net/file/675100This dataset is made available under the Open Database License (http://opendatacommons.org/licenses/odbl/1.0/). The Open Database License (ODbL) is a license agreement intended to allow users to freely share, modify, and use this Dataset while maintaining this same freedom for others, provided that the original source and author(s) are credited.Geoffrey R. Smith, Julio A. Lemos-Espinal
