# Supplementary material for: ﻿Factors related to species richness, endemism, and conservation status of the herpetofauna (Amphibia and Reptilia) of Mexican states
Source: Zookeys. 2022 Apr 20;1097:85–101. doi: 10.3897/zookeys.1097.80424 (PMC9046365; doi:10.3897/zookeys.1097.80424)
Supplement: Supplementary material 1 — Table S1 [file zookeys-1097-085-s001.docx]

**Supplementary Table 1** Alphabetical list of the 27 Mexican States with a comprehensive list published recently. Source refers to the references from which the checklist for each specific state was obtained. Updates list references used to update the original checklist we used for each state.

| **STATE** | **SOURCE** | **UPDATES** |
| --- | --- | --- |
| Aguascalientes | Carbajal-Márquez and Quintero-Díaz 2016 | Cox et al. 2018 |
| Baja California | Grismer 2002; Hollingsworth et al. 2015 | Cox et al. 2018; Meik et al. 2018 |
| Baja California Sur | Grismer 2002 | Cox et al. 2018; Meik et al. 2018 |
| Campeche | González-Sánchez et al. 2017 | Ortíz-Medina et al. 2020; Palacios-Aguilar and Flores-Villela 2020 |
| Chiapas | Johnson et al. 2015b | Hernández-Ordóñez et al. 2017; Clause et al. 2020; Palacios-Aguilar and Flores-Villela 2020 |
| Chihuahua | Lemos-Espinal et al. 2017 | Blair and Hansen 2018; Cox et al. 2018; Palacios-Aguilar and Flores-Villela 2020 |
| Coahuila | Lemos-Espinal and Smith 2016; Lazcano et al. 2019 | Baeza-Tarin et al. 2018 |
| Colima | Lemos-Espinal et al. 2020 | Horowitz 1955; Montanucci 1979; Hillis et al. 1983; Platz 1991; Webb 2001; McCranie and Köhler 2004; Zaldivar-Riverón et al. 2004; Pérez-Ramos and Saldaña-de la Riva 2008; Lavin et al. 2014; Streicher et al. 2014; Cox et al. 2018; Grünwald et al. 2018; O’Connell and Smith 2018; Ramírez-Reyes and Flores-Villela 2018; Montaño-Ravalcaba et al. 2020; Palacios-Aguilar and Flores-Villela 2020; Reyes-Velasco et al. 2020a,b |
| Durango | Lemos-Espinal et al. 2018a, 2019b | Campbell et al. 2018; Caviedes-Solis and Nieto-Montes de Oca 2018 |
| Guerrero | Palacios-Aguilar and Flores-Villela 2018 | Ramírez-Reyes et al. 2017; Campbell et al. 2018; Caviedes-Solis and Nieto-Montes de Oca 2018; Cox et al. 2018; García-Vázquez et al. 2018; Palacios-Aguilar et al. 2018, 2020; Ramírez-Reyes and Flores-Villela 2018; Blancas-Hernández et al. 2019; Grünwald et al. 2019; Köhler et al. 2019; Kaplan et al. 2020; Palacios-Aguilar and Flores-Villela 2020; Palacios-Aguilar and Santos-Bibiano 2020 |
| Hidalgo | Lemos-Espinal and Smith 2015; Lemos-Espinal and Dixon 2016 | Hansen et al. 2016; Badillo-Saldaña et al. 2018; Caviedes-Solis and Nieto-Montes de Oca 2018; Valencia-Herverth et al. 2020 |
| Jalisco | Cruz-Sáenz et al. 2017 | Ramírez-Reyes et al. 2017; Campbell et al. 2018; Caviedes-Solis and Nieto-Montes de Oca 2018; Cox et al. 2018; Ramírez-Reyes and Flores-Villela 2018; Pazos-Nava et al. 2019; Ahumada-Carrillo et al. 2020; Cavazos-Camacho and Ahumada-Carrillo 2020; Palacios-Aguilar and Flores-Villela 2020 |
| Mexico | Lemos-Espinal and Smith 2020d | Campbell et al. 2018; Caviedes-Solis and Nieto-Montes de Oca 2018; Kaplan et al. 2020 |
| Mexico City | Lemos-Espinal and Smith 2020c | García-Alvarado 2016 |
| Michoacán | Alvarado-Díaz et al. 2013 | Mendoza-Hernández and Roth-Monzón 2017; Ramírez-Reyes et al. 2017; Campbell et al. 2018; Cox et al. 2018; Ramírez-Reyes and Flores-Villela 2018; Palacios-Aguilar and Flores-Villela 2020 |
| Morelos | Lemos-Espinal and Smith 2020b | Campbell et al. 2018; Cox et al. 2018; Palacios-Aguilar and Flores-Villela 2020 |
| Nayarit | Woolrich-Piña et al. 2016 | Ramírez-Reyes et al. 2017; Campbell et al. 2018; Cox et al. 2018; Ramírez-Reyes and Flores-Villela 2018; Palacios-Aguilar and Flores-Villela 2020 |
| Nuevo León | Lemos-Espinal et al. 2016; Nevárez de los Reyes et al. 2016 | Nevarez de los Reyes et al. 2019a,b |
| Oaxaca | Mata-Silva et al. 2015 | Gray et al. 2016; Parra-Olea et al. 2016; Campbell et al. 2016, 2018; Canseco-Márquez et al. 2017a,b; Ramírez-Reyes et al. 2017; Caviedes-Solis and Nieto-Montes de Oca 2018; García-Padilla et al. 2019; Mata-Silva et al. 2019 |
| Puebla | Woolrich-Piña et al. 2017 | Caviedes-Solis and Nieto-Montes de Oca 2018; Campbell et al. 2018; Cox et al. 2018; de la Torres-Loranca et al. 2020; Fernández-Badillo et al. 2020; Palacios-Aguilar and Flores-Villela 2020 |
| Querétaro | Dixon and Lemos-Espinal 2010; Cruz-Elizalde et al. 2019 |  |
| Quintana Roo | González-Sánchez et al. 2017 |  |
| San Luis Potosí | Lemos-Espinal et al. 2018b | Guajardo Welsh et al. 2020; Palacios-Aguilar and Flores-Villela 2020 |
| Sinaloa | Lemos-Espinal and Smith 2020a | Campbell et al. 2018; Cox et al. 2018; Trageser and Schell 2018; Loc-Barragán et al. 2020; Palacios-Aguilar and Flores-Villela 2020 |
| Sonora | Rorabaugh 2016; Lemos-Espinal et al. 2019a | Cox et al. 2018; Meik et al. 2018 |
| Tamaulipas | Farr 2015; Terán-Juárez et al. 2016 | Rautsaw et al. 2018; Sosa-Tovar et al. 2019 |
| Yucatán | González-Sánchez et al. 2017 | Palacios-Aguilar and Flores-Villela 2020 |

**References**

Ahumada-Carrillo IT, Grünwald CI, Cuellar MAL, Jones JM (2020) Some records of amphibians and reptiles from the Wixarica Region in the State of Jalisco, Mexico. Herpetological Review 51: 277–281.

Alvarado-Díaz J, Suazo-Ortuno I, Wilson LD, Medina-Aguilar O (2013) Patterns of physiographic distribution and conservation status of the herpetofauna of Michoacan, Mexico. Amphibian and Reptile Conservation 7: 128–170.

Badillo-Saldaña LM, Lara-Tufiño JD, Ramírez-Bautista A, Campbell JA (2018) New state record and potential distribution of the snake *Sibon nebulatus* (Dipsadidae) from Mexico. Western North American Naturalist 78: 242–246. https://doi.org/10.3398/064.078.0201

Baeza-Tarin F, Hernandez T, Giovaneto L, Trevino AE, Lazcano D, Graham SP (2018) Geographic distribution: *Lampropeltis gentilis*. Herpetological Review 49: 505.

Blair RC, Hansen RW (2018) Geographic distribution: *Lampropeltis alterna*. Herpetological Review 49: 718.

Blancas-Hernández JC, Palacios-Aguilar R, Santos-Bibiano R (2019) *Tropidodipsas sartori* (Cope, 1863) (Squamata: Dipsadidae): an addition to the snake fauna from Guerrero, Mexico. Herpetozoa 32: 91–93. DOI 10.3897/herpetozoa.32.e35911

Campbell JA, Brodie ED Jr, Caviedes-Solis IW, Nieto-Montes de Oca A, Luja VH, Flores-Villela O, García-Vázquez UO, Sacher GC, Wostl E, Smith EN (2018) Systematics of the frogs allocated to *Sarcohyla bistincta* sensu lato (Cope, 1877), with description of a new species from western Mexico. Zootaxa 4422: 366–384. https://doi.org/10.11646/zootaxa.4422.3.3

Canseco-Márquez L, Aguilar-López JL, Luría-Manzano R, Pineda E, Caviedes-Solís IW (2017a) A new species of treefrog of the genus *Ptychohyla* (Anura: Hylidae) from southern Mexico. Zootaxa 4317: 279–290. https://doi.org/10.11646/zootaxa.4317.2.5

Canseco-Márquez L, Ramírez-González CG, González-Bernal E (2017b) Discovery of another new species of *Charadrahyla* (Anura; Hylidae) from the cloud forest of northern Oaxaca, México. Zootaxa 4329: 64-72. https://doi.org/10.11646/zootaxa.4329.1.2

Carbajal-Márquez RA, Quintero-Díaz GE (2016) The herpetofauna of Aguascalientes, Mexico. Revista Mexicana de Herpetologie 2: 1–30

Cavazos-Camacho C, Ahumada-Carrillo IT (2020) Geographic distribution: *Pituophis lineatocollis*. Herpetological Review 51: 275.

Caviedes-Solis IW, Nieto-Montes de Oca A (2018) A multilocus phylogeny of the genus *Sarcohyla* (Anura: Hylidae), and an investigation of species boundaries using statistical species delimitation. Molecular Phylogenetics and Evolution 118: 184–193. https://doi.org/10.1016/j.ympev.2017.09.010

Clause AG, Luna-Reyes R, Nieto-Montes de Oca A (2020) A new species of *Abronia* (Squamata: Anguidae) from a protected area in Chiapas, Mexico. Herpetologica 76: 330–343. https://doi.org/10.1655/Herpetologica-D-19-00047

Cox CL, Davis Rubosky AR, Holmes IA, Reyes-Velasco J, Roelke CE, Smith EN, Flores-Villela O, McGuire JA, Campbell JA (2018) Synopsis and taxonomic revision of three genera in the snake tribe Sonorini. Journal of Natural History 52: 945–988. https://doi.org/10.1080/00222933.2018.1449912

Cruz-Elizalde R, Ramírez-Bautista A, Hernández-Salinas U, Berriozabal-Islas C, Wilson LD (2019) An updated checklist of the herpetofauna of Querétaro, Mexico: species richness, diversity, and conservation status. Zootaxa 4638: 273–290. https://doi.org/10.11646/zootaxa.4638.2.7

Cruz-Sáenz D, Muñoz-Nolasco FJ, Mata-Silva V, Johnson JD, García-Padilla E, Wilson LD (2017) The herpetofauna of Jalisco, Mexico: composition, distribution, and conservation. Mesoamerican Herpetology 4: 23–118.

De la Torres-Loranca MA, Martínez-Fuentes RG, Canseco-Marquez L, García-Vázquez UO (2020) New records of amphibians and reptiles from Sierra de Zongolica, Veracruz and Puebla, Mexico. Herpetological Review 51: 550–553.

Dixon JR, Lemos-Espinal JA (2010) Anfibios y Reptiles del Estado de Querétaro, México / Amphibians and Reptiles of the State of Querétaro, Mexico. CONABIO, México, i-xiv + 428 pp.

Farr W (2015) Herpetofauna of Tamaulipas. In: Lemos-Espinal, JA (Ed) Amphibians and Reptiles of the US – Mexico Border States / Anfibios y Reptiles de los Estados de la Frontera México – Estados Unidos. Texas A and M University Press, Texas, 101–121.

Fernández-Badillo L, Morales-Capellán N, González-Bonilla GT, Canseco-Márquez L, Hernández-Silva DA (2020) On the critically endangered Cofre de Perote salamander (*Isthmura naucampatepetl*): discovery of a new population in Puebla, Mexico, and update of its known distribution. Amphibian and Reptile Conservation 14: 200–205.

García-Alvarado F (2016) Geographic distribution: *Hemidactylus frenatus*. Herpetological Review 47: 423.

García-Padilla E, Figiel JJ, Wilson LD, Mata-Silva V (2019) Geographical distribution: *Bolitoglossa mexicana*. Herpetological Review 50: 744.

García-Vázquez UO, Pavón-Vázquez CJ, Blancas-Hernández JC, Blancas-Calva E, Centenero-Alcalá E (2018) A new rare species of the *Rhadinaea decorata* group from the Sierra Madre del Sur of Guerrero, Mexico (Squamata, Colubridae). ZooKeys 780: 137–154. https://doi.org/10.3897/zookeys.780.25593

González-Sánchez VH, Johnson JD, García-Padilla E, Mata-Silva V, DeSantis DL, Wilson LD (2017) The herpetofauna of the Mexican Yucatan Peninsula: composition, distribution, and conservation. Mesoamerican Herpetology 4: 264–380

Gray L, Meza-Lázaro R, Poe S, Nieto-Montes de Oca A (2016) A new species of semiaquatic *Anolis* (Squamata: Dactyloidae) from Oaxaca and Veracruz, Mexico. Herpetological Journal 26: 253–262.

Grismer LL (2002) Amphibians and Reptiles of Baja California, including its Pacific Islands and the Islands of the Sea of Cortés*.* University of California Press, California.

Grünwald CI, Franz-Chávez H, Morales-Flores KI, Ahumada-Carrillo IT, Jones JM (2019) A rare new treefrog of the genus *Sarcohyla* (Anura: Hylidae) from Guerrero, Mexico. Zootaxa 4712: 345–364. https://doi.org/10.11646/zootaxa.4712.3.2

Grünwald CI, Reyes-Velasco J, Franz-Chávez H, Morales-Flores K, Ahumada-Carrillo I, Jones JM, Boissinot S (2018) Six new species of *Eleutherodactylus* (Anura: Eleutherodactylidae: subgenus *Syrrhophus*) from Mexico, with a discussion of their systematic relationships and the validity of related species. Mesoamerican Herpetology 5: 7–81.

Guajardo Welsh R, Lazcano D, Bryson RW (2020) Geographic distribution: *Lampropeltis leonis*. Herpetological Review 51: 80.

Hansen RW, Fernández-Badillo L, Ramírez-Bautista A, Avalos-Torales O (2016) Geographic distribution: *Lampropeltis mexicana*. Herpetological Review 47: 262–263.

Hernández-Ordóñez O, Cervantes-López M de J, González-Hernández A, Andresen E, Reynoso VH (2017) First record of the limestone rainfrog *Craugastor psephosypharus* (Amphibia: Anura: Craugastoridae) in Mexico. Revista Mexicana de Biodiversidad 88: 260–264. https://doi.org/10.1016/j.rmb.2017.01.005

Hillis DM, Frost JS, Wright DA (1983) Phylogeny and biogeography of the *Rana* *pipiens* complex: a biochemical evaluation. Systematic Biology 32: 132–143. https://doi.org/10.1093/sysbio/32.2.132

Hollingsworth BD, Mahrdt CR, Grismer LL, Lovich RE (2015) Herpetofauna of Baja California. In: Lemos-Espinal JA (Ed) Amphibians and Reptiles of the US – Mexico Border States / Anfibios y Reptiles de los Estados de la rontera México – Estados Unidos, Texas A and M University Press, Texas, 15–33.

Horowitz SB (1955) An arrangement of the subspecies of the horned toad, *Phrynosoma orbiculare* (Iguanidae). American Midland Naturalist 54: 204–218. https://doi.org/10.2307/2422188

Johnson JD, Mata-Silva V, García-Padilla E, Wilson LD (2015) The herpetofauna of Chiapas, Mexico: composition, physiographic distribution, and conservation status. Mesoamerican Herpetology 2: 272–329.

Kaplan M, Heimes P, Aguilar R (2020) A new species of *Sarcohyla* (Anura: Hylidae: Hylini) from the Sierra Madre del Sur of Guerrero and Estado de México, México. Zootaxa 4743: 382–390. https://doi.org/10.11646/zootaxa.4554.2.3

Köhler G, Petersen CBP, Méndez de la Cruz FR (2019) A new species of anole from the Sierra Madre del Sur in Guerrero, Mexico (Reptilia, Squamata, Dactyloidae: *Norops*). Vertebrate Zoology 69: 145–160.

Lavin PA, Lazcano D, Gadsden H (2014) Anfibios y reptiles exóticos y traslocados invasores. In: Mendoza R, Koleff P (Eds) Especies Acuáticas Invasoras en México. Comisión Nacional para el Conocimiento y Uso de la Biodiversidad, México, 435–441.

Lazcano D, Nevárez-de los Reyes M, García-Padilla E, Johnson JD, Mata-Silva V, DeSantis DL, Wilson LD (2019) The herpetofauna of Coahuila, Mexico: Composition, distribution, and conservation status. Amphibian and Reptile Conservation 13: 31–94.

Lemos-Espinal JA, Dixon JR (2016) Anfibios y Reptiles de Hidalgo / Amphibians and Reptiles of Hidalgo*.* CONABIO, México.

Lemos-Espinal JA, Smith GR (2015) Amphibians and reptiles of the state of Hidalgo, Mexico. Check List 11: 1642. https://doi.org/10.15560/11.3.1642

Lemos-Espinal JA, Smith GR (2016) Amphibians and reptiles of the state of Coahuila, Mexico, with comparisons with adjoining states. ZooKeys 593: 117–137. doi: 10.3897/zookeys.593.8484

Lemos-Espinal JA, Smith GR (2020a) A checklist of the amphibians and reptiles of Sinaloa, Mexico with a conservation status summary and comparisons with neighboring states. ZooKeys 931: 85–114. doi: 10.3897/zookeys.931.50922

Lemos-Espinal JA, Smith GR (2020b) A conservation checklist of the herpetofauna of Morelos, with comparisons with adjoining states. ZooKeys 941: 121–144. doi: 10.3897/zookeys.941.52011

Lemos-Espinal JA, Smith GR (2020c) A conservation checklist of the amphibians and reptiles of Mexico City, with comparisons with adjoining states. ZooKeys 951: 109–131. doi: 10.3897/zookeys.951.52578

Lemos-Espinal JA, Smith GR (2020d) A conservation checklist of the amphibians and reptiles of the State of Mexico, Mexico with comparisons with adjoining states. ZooKeys 953: 137–159. doi: 10.3897/zookeys.953.50881

Lemos-Espinal JA, Smith GR, Cruz A (2016) Amphibians and reptiles of the state of Nuevo Léon, Mexico. ZooKeys 594: 123–141. doi: 10.3897/zookeys.594.8289

Lemos-Espinal JA, Smith GR, Rorabaugh JC (2019a) A conservation checklist of the amphibians and reptiles of Sonora, Mexico, with updated species lists. ZooKeys 829: 131–160. doi: 10.3897/zookeys.829.32146

Lemos Espinal JA, Smith GR, Valdez-Lares R (2019b) Amphibians and Reptiles of Durango, México. ECO-Herpetological Publishing and Distribution, Arizona.

Lemos-Espinal JA, Smith GR, Woolrich-Piña GA (2018b) Amphibians and reptiles of the state of San Luis Potosí, Mexico, with comparisons with adjoining states. ZooKeys 753: 83–106. doi: 10.3897/zookeys.753.21094

Lemos-Espinal JA, Smith GR, Pierce LJS, Painter CW (2020) The amphibians and reptiles of Colima, Mexico, with a summary of their conservation status. ZooKeys 927: 99–125. doi: 10.3897/zookeys.927.50064

Lemos-Espinal JA, Smith GR, Woolrich-Piña GA, Cruz A (2017) Amphibians and reptiles of the state of Chihuahua, Mexico, with comparisons with adjoining states. ZooKeys 658: 105–130. doi: 10.3897/zookeys.658.10665

Lemos-Espinal JA, Smith GR, Gadsden-Esparza H, Valdez-Lares R, Woolrich-Piña GA (2018a) Amphibians and reptiles of the state of Durango, Mexico, with comparisons with adjoining states. ZooKeys 748: 65–87. doi: 10.3897/zookeys.748.22768

Loc-Barragán JA, Franz-Chávez H, Grünwald AJ, Grünwald CI (2020) Geographic distribution: *Eleutherodactylus palidus*. Herpetological Review 51: 532.

Mata-Silva V, Johnson JD, Wilson LD, García-Padilla E (2015) The herpetofauna of Oaxaca, Mexico: composition, physiographic distribution, and conservation status. Mesoamerican Herpetology 2: 6–62.

Mata-Silva V, Rocha A, Ramírez-Bautista A, Berriozabal-Islas C, Wilson LD (2019) A new species of forest snake of the genus *Rhadinaea* from tropical montane rainforest in the Sierra Madre del Sur of Oaxaca, Mexico (Squamata, Dipsadidae). ZooKeys 813: 55–65. doi: 10.3897/zookeys.813.29617

McCranie JR, Köhler G (2004) *Laemanctus longipes*. Catalogue of American Amphibians and Reptiles 795: 1–4.

Meik JM, Schaack S, Flores-Villela O, Streicher JW (2018) Integrative taxonomy at the nexus of population divergence and speciation in insular speckled rattlesnakes. Journal of Natural History 52: 989–1016. https://doi.org/10.1080/00222933.2018.1429689

Mendoza-Hernández AA, Roth-Monzón AJ (2017) Geographic distribution: *Trachemys ornata*. Herpetological Review 48: 387–388.

Montaño-Ravalcaba C, Reyes-Velasco J, Grünwald CI, Jones JM (2020) Geographic distribution: *Geophis pyburni*. Herpetological Review 51: 545.

Montanucci RR (1979) Notes on systematics of horned lizards allied to *Phrynosoma orbiculare* (Lacertilia: Iguanidae). Herpetologica 35: 116–124.

Nevarez de los Reyes M, Lazcano D, Wilson LD (2019a) Geographic distribution: *Gerrhonotus ophiurus*. Herpetological Review 50: 328.

Nevarez de los Reyes M, Lazcano D, Centenero-Alcalá E, Eryan-Sánchez-Morales N, Serra-Ortíz MA, Wilson LD (2019b) Geographic distribution: *Boa imperator*. Herpetological Review 50: 528.

Nevárez de los Reyes M, Lazcano D, García-Padilla E, Mata-Silva V, Johnson JD, Wilson LD (2016) The herpetofauna of Nuevo León, Mexico: composition, distribution, and conservation. Mesoamerican Herpetology 3: 558–638.

O’Connell KA, Smith EN (2018) The effect of missing data on coalescent species delimitation and a taxonomic revision of whipsnakes (Colubridae: *Masticophis*). Molecular Phylogenetics and Evolution 127: 356–366. https://doi.org/10.1016/j.ympev.2018.03.018

Ortiz-Medina JA, Cabrera-Ceri DI, Nahuat-Cervera PE, Chablé-Santos JB (2020) New distributional records from the herpetofauna of Campeche and Yucatán, Mexico. Herpetological Review 51: 83–87.

Palacios-Aguilar R, Flores-Villela O (2018) An updated checklist of the herpetofauna from Guerrero, Mexico. Zootaxa 4422: 1–24. https://doi.org/10.11646/zootaxa.4422.1.1

Palacios-Aguilar R, Flores-Villela O (2020) Taxonomic revision and comments on two groups of the genus *Coniophanes* (Squamata: Dipsadidae). Vertebrate Zoology 70: 111–124.

Palacios-Aguilar R, Santos-Bibiano R (2020) A new species of direct-developing frog of the genus *Eleutherodactylus* (Anura: Eleutherodactylidae) from the Pacific lowlands of Guerrero, Mexico. Zootaxa 4750: 250–260.

Palacios-Aguilar R, Santos-Bibiano R, Flores-Villela O (2018) A new species of *Lepidophyma* (Squamata: Xantusiidae) from the Pacific lowlands of Guerrero, Mexico. Journal of Herpetology 52: 327–331. https://doi.org/10.1670/17-061

Parra-Olea G, Rovito SM, García-París M, Maisano JA, Wake DB, Hanken J (2016) Biology of tiny animals: three new species of minute salamanders (Plethodontidae: *Thorius*) from Oaxaca, Mexico. PeerJ 4: e2694. https://doi.org/10.7717/peerj.2694

Pazos-Nava FN, Álvaro-Montego RI, Capul-Magaña FG, de Quevedo-Machain RG, Flores-Guerro US, Velasco JA, Escobedo-Galván AH (2019) First verified record of *Anolis sagrei* Cocteau in Duméril and Bibron, 1837 from the central Pacific Coast of Mexico. BioInvasions Records 8: 568–574. https://doi.org/10.3391/bir.2019.8.3.12

Pérez-Ramos E, Saldaña-de La Riva L (2008) Morphological revision of lizards of the *formosus* group, genus *Sceloporus* (Squamata: Sauria) of southern México, with description of a new species. Bulletin of the Maryland Herpetological Society 44: 77–97.

Platz JE (1991) *Rana berlandieri* Baird, Rio Grande Leopard Frog. Catalogue of American Amphibians and Reptiles 508: 1–4.

Ramírez-Reyes T, Flores-Villela O (2018) Taxonomic changes and description of two new species from the *Phyllodactylus lanei* complex (Gekkota: Phyllodactylidae) in Mexico. Zootaxa 4407: 151–190. https://doi.org/10.11646/zootaxa.4407.2.1

Ramírez-Reyes T, Piñero D, Flores-Villela O, Vázquez-Domínguez E (2017) Molecular systematic, species delimitation and diversification patterns of the *Phyllodactylus lanei* complex (Gekkota: Phyllodactylidae) in Mexico. Molecular Phylogenetics and Evolution 115: 82–94. https://doi.org/10.1016/j.ympev.2017.07.008

Rautsaw RM, Holding ML, Strickland JL, Castañeda Gaytán JJ, García González FC, Castañeda Gaytán JG, Borja Jiménez JM, Parkinson CL (2018) Geographic distribution: *Hypsiglena tanzeri*. Herpetological Review 49: 287.

Reyes-Velasco JR, Grünwald CI, Jones JM, Ahumada Carrillo IT (2020b) Una revisión de la herpetofauna de Colima, México. Revista Latinoamerica de Herpetologia 3: 61–82.

Reyes-Velasco J, Adams RH, Boissinot S, Parkinson CL, Campbell JA, Castoe TA, Smith EN (2020a) Genome-wide SNPs clarify lineage diversity confused by coloration in coralsnakes of the *Micrurus diastema* species complex (Serpentes: Elapidae). Molecular Phylogenetics and Evolution 147: 106770. https://doi.org/10.1016/j.ympev.2020.106770

Rorabaugh JC, Lemos-Espinal JA (2016) A Field Guide to the Amphibians and Reptiles of Sonora, Mexico. ECO Herpetological Publishing and Distribution, Arizona.

Sosa-Tovar GH, García-Padilla E, Wilson LD, Mata-Silva V (2019) Geographic distribution: *Hemidactylus mabouia*. Herpetological Review 50: 746.

Streicher JW, Devitt TJ, Goldberg CS, Malone JH, Blackmon H, Fujita MK (2014) Diversification and asymmetrical gene flow across time and space: lineage sorting and hybridization in polytypic barking frogs. Molecular Ecology 23: 3273–3291. https://doi.org/10.1111/mec.12814

Terán-Juàrez SA, García-Padilla E, Mata-Silva V, Johnson JD, Wilson LD (2016) The herpetofauna of Tamaulipas, Mexico: Composition, distribution, and conservation status. Mesoamerican Herpetology 3: 43–113.

Trageser SJ, Schell RA (2018) Geographic distribution: *Marisora brachypoda*. Herpetological Review 49: 504.

Valencia-Herverth R, Fernández-Badillo L, Valencia-Herverth J (2020) Geographic distribution: *Terrapene mexicana*. Herpetological Review 51: 537–538.

Webb RG (2001) Frogs of the *Rana tarahumarae* group in western Mexico. In: Johnson JD, Webb RG, Flores-Villela O (Eds) Mesoamerican Herpetology: Systematics, Zoogeography and Conservation. University of Texas at El Paso, Texas, 20–43.

Woolrich-Piña GA, García-Padilla E, DeSantis DL, Johnson JD, Mata-Silva V, Wilson LD (2017) The herpetofauna of Puebla, Mexico: composition, distribution, and conservation status. Mesoamerican Herpetology 4: 791–884

Woolrich-Piña GA, Ponce Campos P, Loc-Barragán J, Ramírez-Silva JP, Mata-Silva V, Johnson JD, García-Padilla E, Wilson LD (2016) The herpetofauna of Nayarit, Mexico: composition, distribution, and conservation. Mesoamerican Herpetology 3: 376–448.

Zaldivar-Riverón A, León-Regagnon V, Nieto-Montes de Oca A (2004) Phylogeny of the Mexican coastal leopard frogs of the *Rana berlandieri* group based on mtDNA sequences. Molecular Phylogenetics and Evolution 30: 38–49. https://doi.org/10.1016/S1055-7903(03)00141-6
